# Axial and Radial Compression Behavior of Composite Rocket Launcher Developed by Robotized Filament Winding: Simulation and Experimental Validation

**DOI:** 10.3390/polym13040517

**Published:** 2021-02-09

**Authors:** Rajesh Mishra, Bijoy Kumar Behera, Sayan Mukherjee, Michal Petru, Miroslav Muller

**Affiliations:** 1 Department of Material Science and Manufacturing Technology, Faculty of Engineering, Czech University of Life Sciences Prague, Kamycka 129, 165 00 Prague, Czech Republic; muller@tf.czu.cz; 2 Department of Textile & Fiber Engineering, Indian Institute of Technology Delhi, Delhi 110016, India; bijoy.behera@yahoo.com (B.K.B.); mukherjee.sayan.iitd@gmail.com (S.M.); 3 Institute of Nanomaterials, Advanced Technologies and Innovation (CXI), Technical University of Liberec, 461 17 Liberec, Czech Republic; michal.petru@tul.cz

**Keywords:** carbon fibers, glass fibers, mechanical properties, finite element analysis (FEA), filament winding

## Abstract

The principal objective of the work is to compare among carbon-glass filament wound epoxy matrix hybrid composites with a different fiber ratio made by robotized winding processes and optimize the geometry suitable for the Rocket Propelled Grenade Launcher. ANSYS based finite element analysis was used to predict the axial as well as radial compression behavior. Experimental samples were developed by a robot-controlled filament winding process that was incorporated with continuous resin impregnation. The experimental samples were evaluated for the corresponding compressional properties. Filament wound tubular composite structures were developed by changing the sequence of stacking of hoop layers and helical layers, and also by changing the angle of wind of the helical layers while keeping the sequence constant. The samples were developed from carbon and glass filaments with different carbon proportions (0%, 25%, 50%, 75%, and 100%) and impregnated with epoxy resin. The compressional properties of the tubular composites that were prepared by filament winding were compared with the predicted axial and radial compressional properties from computational modelling using the finite element model. A very high correlation and relatively small prediction error was obtained.

## 1. Introduction

Textile reinforced composites can be produced through numerous methods, depending on the applications. ‘Filament Winding’ is one such technique. It has proven to be technically effective and cost competitive over the last few decades [1]. Filament winding is used in different applications in order to make axis symmetrical composite parts, e.g., sewage or supply piping systems, high-pressure vessels, water storage tanks, aircraft fuselage sections, transmission shafts, fishing rods, golf club shafts, etc. This technique is also used in axis-nonsymmetrical parts, like wind turbine blades, chassis in buses, etc. [2,3]. In this process, the continuous filaments are wound over a rotating mandrel in order to produce cylindrical profiles with advanced mechanical performance. Successive layers can be added with different winding angles onto the different profiles of mandrel, in adjacent bands or in repeating patterns that cover the surface of the mandrel with a high degree of uniformity [4,5,6,7]. Mandrel and delivery head movement is controlled in a synchronized way that regulates the fiber/filament path and helps in making the desired pattern. The mechanical properties of filament wound components can be improved by controlling the winding pattern, ratio of the matrix and fibrous material mixture, tension in fiber, and other process variables [8,9,10].

Besides the winding approach, there are other classification criteria that explain the majority of the winding methods available. The nature of winding is additionally affected by the number of spatially turning axes used to wrap the fiber around the mandrel [11,12,13]. All of these criteria allow for procedural differentiation and the allocation of typical topological appearances to the different composite components produced.

**Figure 1:**
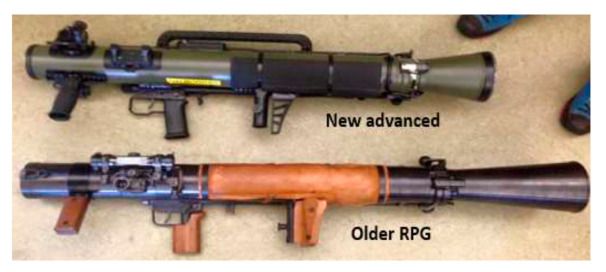
Old and new rocket-propelled grenades (RPG).

The Rocket-Propelled Grenade, which is commonly known by its initials “RPG”, is one of the most lethal weapons used in modern military combat. Today’s Rocket-Propelled Grenades (RPGs)—sometimes called rocket launchers—are frequently used to destroy military vehicles, notably armored personnel carriers. In recent past, RPGs were used to target heavily armored tanks. Militaries around the world define an RPG as a hand-held anti-tank weapon that fires an unguided rocket armed with an explosive warhead. The RPG has proven popular in modern warfare, where enemy combatants use them for destroying powerful tanks and military bases [14,15,16,17,18,19]. Current models of RPGs provide an inexpensive means of delivering an explosive warhead. The accuracy of aiming the target can be a problem because the rockets fired from RPGs are not guided. Rockets from RPGs achieve the best results when fired at close ranges. This often requires people using an RPG to put themselves in the line of fire in order to score an accurate hit. Figure 1 shows representative old and new RPGs.

The filament wound hollow composites can prove to be a suitable solution in developing modern RPGs with reduced weight and enhanced performance. However, the adequate evaluation of their performance in both the radial and axial direction is mandatory, so as to ascertain their applicability in rocket launchers. During the rocket launching, tremendous force is exerted on the launcher in both the longitudinal as well as radial direction [15,16,17]. The launcher experiences expansion in the longitudinal and radial direction. There is a reaction force that causes compression axially and radially. The evaluation of longitudinal and radial expansion in tubular composites of relatively high diameter in rather more complicated. Thus, the compressional properties provide a reasonable explanation of their mechanical performance.

There are various experimental, analytical, and computational methods for evaluating the mechanical properties of composite materials [20,21,22,23]. The finite element method (FEM) is the one of the numerical methods that are more powerful in their application in real world problems and can be used to calculate elastic properties. The commercial software ANSYS (Canonsburg, PA, US) is very user friendly and easy to design the required model. The Rule of Mixtures, based on the mechanics of materials approach, is the most basic model available for prediction of the composite properties from the properties of the matrix material, the fiber material, and the fiber volume fraction [24]. It does not take the fiber shape or the fiber distribution into account. The models assume perfect bonding between the matrix material and fiber material. The matrix material and fiber material are assumed to be orthotropic and they can be simplified to the isotropic case.

The principal objective of current research is to compare among carbon-glass reinforced epoxy matrix hybrid hollow composites with different fiber ratios made by robotized filament winding processes, and to find out the optimum composition and geometry that are suitable for the Rocket Propelled Grenade Launcher. The novelties of the current work include:

- Finite element modeling of the cylindrical RPG by the computer-aided simulation using ANSYS software. The prediction of the axial and radial compression properties of the tubular composites.
- Preparation of the filament wound hybrid tubular composite structures by robotized filament winding machine using carbon and glass tows in different proportions.
  ○ By changing the angle of wind of the helical layers keeping the stacking sequence constant.
  ○ By changing the sequence of stacking of layers, i.e., hoop layers and helical layers at a constant angle of wind.
- Continuous impregnation of filaments with epoxy resin during the winding process.
- Comparison of the prepared tubular composite samples with respect to their axial and radial compression properties and the validation of FEM based prediction of compression properties with the experimental results.

## 2. Materials and Methods 

### 2.1. Materials

In this work, two types of industrial grade filaments were used for preparing the reinforcement in the production of composites:

- Carbon fiber: Torayca Grade T600B-12k-50B (Toray, Chuo City, Tokyo, Japan). This is a high-performance carbon fiber made from PAN (Polyacrylonitrile) precursor.
- The glass fiber is E-Glass grade T600-6k, (Hexcel, Les Avenieres, France). This fiber is also a high-performance fiber and compatible with a different range of resins.

These filaments wound in different proportions were impregnated with Epoxy resin Araldite LY556 along with Anhydride Hardener HY 556 and Accelerator DY 070 for adjusting the reactivity of the system.

Table 1 provides the physical properties of used fibers and resin.

### 2.2. Winding Machine Description

The robotized winding machine consists of two important parts: 1. Headstock with mandrel holder and 2. Movable assembly with creel mounted on it. 

**Figure 2:**
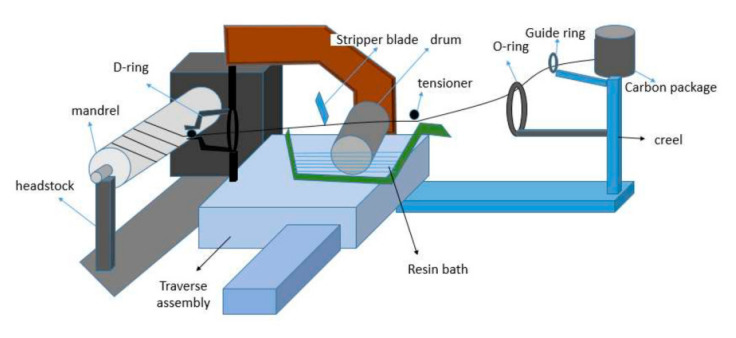
Schematic diagram of the fiber path in the robotized filament winding machine.

The headstock consists of a rotating unit, attached with a three-jaw holding vice. The mandrel of pre-determined size can be tightly mounted on the jaw, and the other end of the mandrel is mounted onto a rotary vice. The rotational speed of the rotary vice is variable and it can be changed according to the angle of wind or type of wind. The movable assembly consists of creel and resin impregnating systems that are joined by a link rod [25,26,27,28]. The creel has six places (three on each side) for the filament packages to be mounted. The rotation of the package holders can be controlled by a friction type tensioning system. The filaments are guided through a guide ring to the O-ring. Two or more filament tows are pulled together in order to achieve the desired bandwidth of the filament bands. The bandwidth is adjusted according to the speed of production. After the creel zone, there comes a shallow bath where the resin is kept and, over that, a polished steel drum is kept partially immersed into the resin bath. As the filament tow passes over the roller, it drags the roller, which results in a rotational movement of the drum. The resin that is up-taken by the surface of the drum applies onto the filament. Now, the filament tow is passed through several stripper knives and comb or separator. The resin-impregnated filament is then passed through a D-ring, which can rotate on its central axis and fix the filament to the right position according to the preset angle of the wind. The whole assembly can move in the parallel direction of the mandrel rotational axis [29,30]. The whole machine can move in five axes: Resin carriage assembly movement in X-axis, D-ring propagation in Y-axis, Height adjustment of the carriage in Z-axis, Rotation of the mandrel in A-axis, and Rotation of the D-ring in B-axis. Figure 2 shows the schematic of the robotized winding machine.

The other technical specifications of the machine are: Maximum Winding Diameter: Ø 400 mm, Maximum Winding Length: 2000 mm, Maximum Rotating weight (Job + Mandrel): 100 kgs, Number of axes (Simultaneous Controls): 4, Number of spindles: 1, Creel Stand: Internal unwinding creel stand of six spools.

### 2.3. Preparation of Tubular Composite Samples

The filaments of Glass and Carbon that are pre-impregnated with the resin during the winding process were wound onto the mandrel, as shown in Figure 2. After the desired width was achieved, the impregnated tube was removed from the mandrel. It was then cured for 180 min. at 120 °C. A similar procedure was followed for all composite samples with different fiber proportion and varying winding angles, as well as winding patterns.

In total, six carbon-glass filament wound composite samples were manufactured. Three samples were prepared with straight cylindrical shape, and three other samples with tapering. The dimensions and specifications of the filament wound composites are: Length: 980 mm, Outer diameter: 90 mm, Inner diameter: 84 mm, Number of layers: 5, Angle of wind: Helical (30, 45, 55 degrees), Hoop (90 degrees), and Tapering angle: 1°.

The helical layers of the filament wound samples were altered between three angles. The sequence of the layers was kept constant for all of the samples. The number of layers was also kept constant in order to compare the compressional behavior according to change in angle of wind.

Table 2 provides the details of the composite samples.

**Figure 3:**
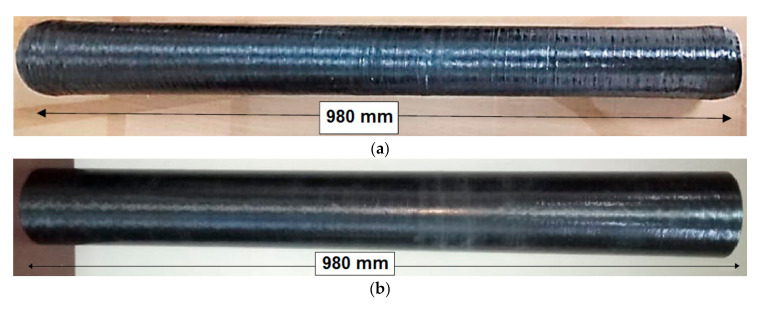
Sample of composite tubes (**a**) cylindrical and (**b**) tapered at 1° angle.

Sample composite tubes are shown in Figure 3.

**Figure 4:**
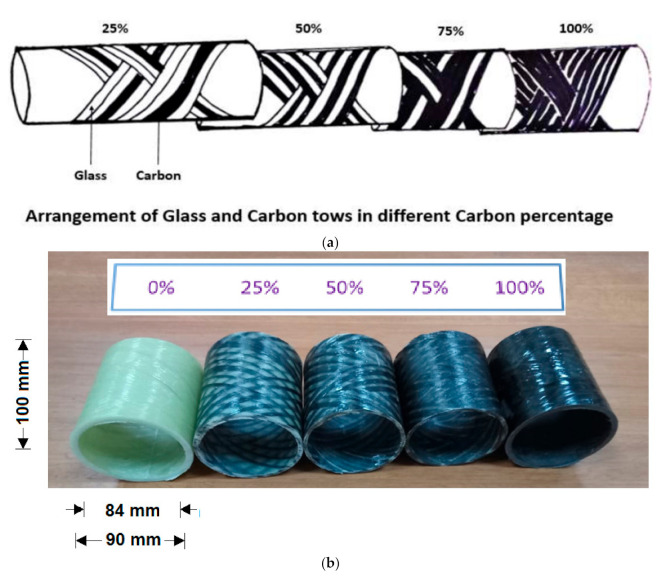
(**a**) Arrangement of glass and carbon tows and (**b**) composite samples with different carbon content (0%, 25%, 50%, 75%, and 100%).

Additionally, cylindrical samples were prepared by helical winding with different carbon content (0%, 25%, 50%, 75%, and 100%), as shown in Figure 4.

### 2.4. Testing Standards & Testing Methods

#### 2.4.1. Radial Compressional Behavior

The compressional properties of the cylindrical samples were tested on the universal testing instrument INSTRON (Norwood, MA, US), according to ASTM D2412-10. The radial compression tests of specimen cylinders were performed between two flat platens on a testing machine (Instron 3365) at a crosshead speed of 10 mm/min. [31]. The load plates were set parallel to each other before testing, and all of the cylinders were compressed until limited crush. The final values were the averages of ten measurements. 

#### 2.4.2. Axial Compressional Behavior

The axial compression testing of specimen cylinders was performed between two flat plates on a testing machine (Instron 3365) according to ASTM D2412-11 at a crosshead speed of 2 mm/min. [32]. All of the cylinders were limited to the ultimate crush, but, due to smaller length to radius ratio, the insufficient length results in the edge crushing of the samples. 

### 2.5. Modeling with ANSYS 

The related physical properties of the reinforcing fibers and resin were tested and then the physical properties of the composite were calculated while using rule of mixture and the Halpin–Tsai model. The Halpin–Tsai model is commonly used to predict the effective compressional strength and modulus for continuous fiber reinforced composites with perfect fiber alignment as reported by several researchers [33,34,35]. The details of derivation for the Halpin–Tsai equations are reported in the review that was written by Halpin Affdl and Kardos [34]. The Halpin–Tsai equation has the following form:

(1) $$K_{c}=K_{m}\left[\frac{1+\xi \zeta V_{f}}{1-\eta V_{f}}\right]$$

(2) $$\mathrm{With}\;\eta =\left[\frac{(K_{f}/K_{m})-1}{(K_{f}/K_{m})+\zeta }\right]$$

where *K_c_* represents the effective compressional property of the composite, while *K_f_* and *K_m_* are the corresponding fiber and matrix compressional properties, *V_f_* denotes the fiber volume fraction, and *ζ* is a geometrical parameter, which represents the reinforcement geometry, packing geometry, and loading conditions. In the present analysis, the geometry is defined by the winding angle, tapering, and the winding pattern.

Three-dimensional models of filament wound composites, fractional, and continuous structure were developed. In a fractional model, the wound tow (continuous filament of fiber) was considered as a tape of definite width that was wrapped around the core that takes the effect of gap between the layers and distance between the consecutive widths into consideration. This helps in a better simulation of the mechanical properties at the micro level. In continuous model, the tow wound over the mandrel is treated as a uniform ply sheet of filaments that are oriented at a defined angle on the core. This provides a more accurate picture at the macro level. The developed model was imported to ANSYS platform in order to simulate the model, so as to calculate the stress and internal pressure using the Finite Element Method (FEM). 

**Figure 5:**
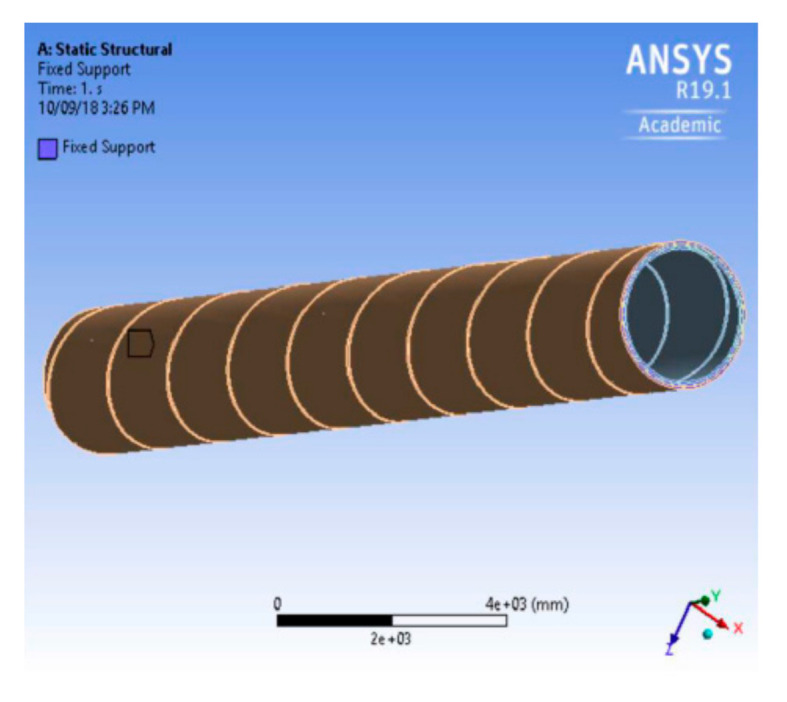
Simulation of plies in ANSYS.

Static structural tools were used from ANSYS and corresponding Engineering data of the fiber (e.g., carbon, glass, etc.) and resin (e.g., Epoxy) were imported. After importing geometry, the contact regions (e.g., bonded, rough, and frictionless) between different layers that are wound on different angles were identified and defined. The mesh module contains tools that allow for generating meshes on parts and assemblies created. In addition, the mesh module contains functions that verify an existing mesh. Figure 5 shows the simulation of plies on ANSYS.

In the final step for performing the simulation, the job module was used to create, analyze, and view a basic plot of the results. Certain boundary conditions were defined, and adaptive analyses and co-executions were also performed. The assembling of different layers was simulated on the cylindrical profile by defining fiber orientation in each layer. The number of layers and thickness of layers can also be varied. 

The optimization module was used to create the topology or shape of the model with a set of defined boundary conditions. The input parameters (e.g., fiber properties, winding angle, internal diameter, external diameter, etc.) were given and output parameters (e.g., maximum equivalent compression stress, total maximum compressional deformation) were obtained. 

## 3. Results and Discussion

### 3.1. Simulation of Radial and Axial Compression

The simulation model of layered cylindrical sample was designed, and the analysis was carried out using ANSYS Workbench. The main advantages of simulation are to study the behavior of a system without building it. The rsults are accurate in general, as compared to analytical models. This helps to analyze unexpected phenomenon and behavior of the system. 

A rocket launcher is used to propel the rocket with very high energy. After the launching, there is a reaction force exerted axially and radially, which is compressive in nature. Thus, the radial and axial compressive stress and modulus are the essential parameters that must be predicted, so as to ensure the performance and longevity of a tubular composite used in this purpose.

**Figure 6:**
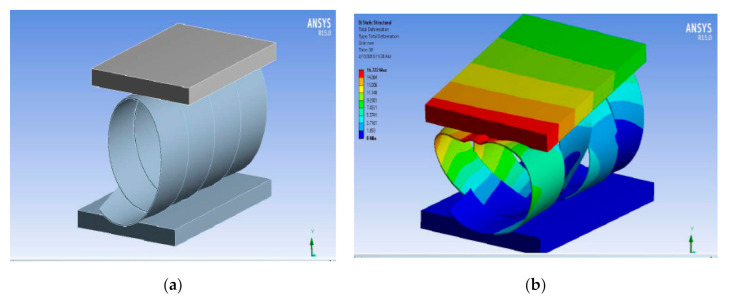
The simulation of radial compression for one layer (**a**) Basic pattern, (**b**) Total deformation.

Figure 6 shows the simulation of radial compression.

From the simulation, the maximum radial stress and compressive modulus were predicted. It also indicates the stress distribution and stress concentration. The maximum load was be predicted for all the samples with varying Carbon:Glass proportion. Based on the optimum results, the prediction was also carried out for a different winding angle and winding pattern for the best fiber composition.

**Figure 7:**
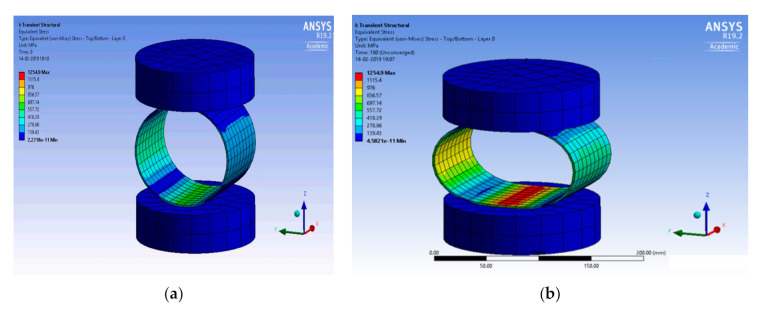
Simulation of radial stress (**a**) Initial stress and (**b**) Peak stress.

Figure 7 shows the simulation of radial stress.

Based on the input material properties, the simulation of peak stress was carried out for the five layered tubular composite tubes. The simulation indicated the maximum compression stress and strain from which the modulus was predicted. Further analysis was carried out for this composition with a different angle and pattern of winding, as the 25% Carbon and 75% Glass baaed sample produced the highest compressive load.

**Figure 8:**
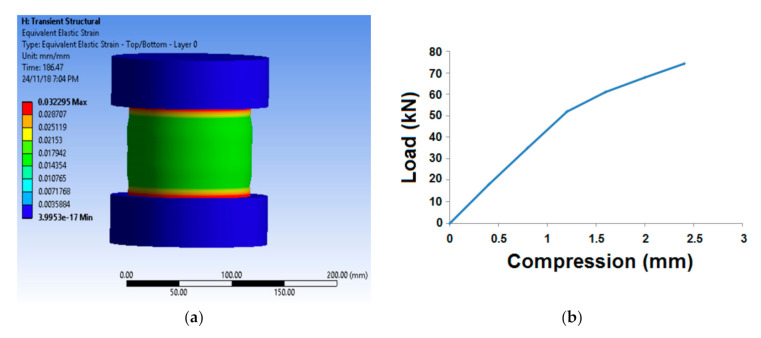
(**a**) Simulation of axial compression and (**b**) A typical load- compression curve.

The longitudinal reaction force after rocket launching leads to substantial compression in the axial direction. Therefore, it is essential to predict and evaluate the axial compression parameters, e.g., compressive stress, maximum strain, and modulus. Figure 8 shows a representative simulation behavior of axial compression and the typical load- compression curve.

From the simulation, the maximum equivalent stress and strain were predicted. The maximum stress was obtained for sample (25% carbon, 75% glass). The corresponding equivalent strain values were obtained, and the modulus was calculated. Figure 8b, which shows the nature of compression load with respect to strain, shows a typical load-compression curve. The curve shows two distinct slopes indicating the elastic and inelastic deformation during axial compression. Several other researchers also reported similar behavior [2,3,27].

### 3.2. Experimental Validation of Radial and Axial Compression

Compression testing in both radial and axial direction was performed according to ASTM D2412-10 & 11 in order to test the compressive strength and modulus of the filament wound composite structures.

#### 3.2.1. Radial Compression Behavior

**Figure 9:**
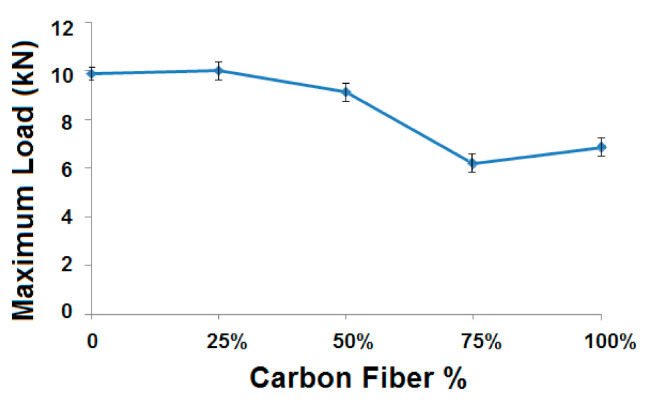
Radial compression load for different samples with varying carbon fiber%.

Figure 9 shows the radial compression load measured for different samples with varying carbon fiber%.

It can be observed that the sample (25% carbon, 75% glass) is having maximum radial compressive load, and the sample (75% carbon, 25% glass) is having the lowest radial compressive load bearing capability. Among the hybrid composite samples, the behaviors are dependent on the properties of the component fibers, as shown in Table 1, and their combinations, as per the Halpin–Tsai model [34,35]. The compressional strength of glass filament tow is higher than that of carbon tow. Because of this, a higher proportion of glass fibers may lead to higher compression load [25]. It might be noted that the sample (25% carbon, 75% glass) shows a compressional load slightly higher than the 100% glass-based sample. It can be due to the fact that carbon fiber tows have lower density than the glass fiber tow, as shown in Table 1. Thus, the carbon fibers occupy a higher volume for the same linear density. The addition of carbon fiber up to 25% increases the contact surface and, thus, a higher interfacial area with the matrix. This could be the reason for slightly increased compressional performance in the sample with (25% carbon, 75% glass). Of course, this increment is only marginal and not very significant. With a further increase in carbon fiber proportion, the inferior fiber compressive strength as compared to glass fiber tow comes into picture and the performance is observed to deteriorate with an increasing carbon fiber percentage. Another deviation is observed for the 100% carbon fiber-based sample, which shows a higher compressive load when compared to sample (75% carbon, 25% glass). This improvement might be attributed to the maximum cohesiveness of whole carbon tows (in the absence of any glass tow), which leads to stronger inter-fiber bonding and, thus, an enhanced mechanical performance [21,25]. However, this cohesiveness is not particularly significant for dominating over the inherent fiber properties in the sample with (50% carbon, 50% glass).

**Figure 10:**
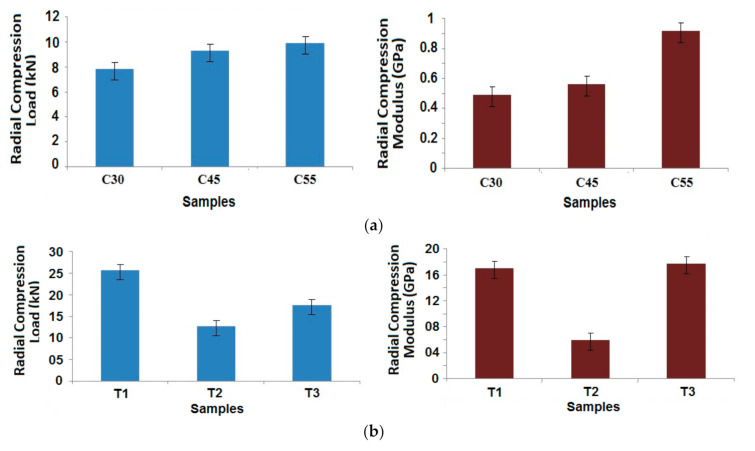
Radial compression behavior of (**a**) Cylindrical composite samples at different angles of wind and (**b**) Tapered composite samples for different patterns of wind.

Based on the above results, it was determined to study the radial compressional properties of the best performing samples (25% carbon, 75% glass) with different angles of wind and patterns of winding in the case of tapered samples. The results are shown in Figure 10a,b for the cylindrical and tapered composite samples, respectively.

A simple trend was observed in the case of the cylindrical samples, as the winding angle increases, radial compression properties improve [2,3,4]. This can be attributed to the positioning of the fibers in the load bearing direction. Based on Halpin–Tsai model, the geometrical parameter is dependent on the angle of wind. A higher angle of wind helps in increasing the contribution towards radial compressional performance [35]. However, with respect to dimensional stability and a balanced structure, 45° winding angle is the most preferable. 

Maximum compressional load was observed in sample T1, which is constructed with alternating layers of winding and hooping. This results in a compact structure with superior performance. Sample T2 shows the lowest compression load, owing to the middle layers with successive hooping pattern, which may not be able to provide enough bonding with the matrix. The compressional modulus is relatively higher in case of both T1 and T3 in contrast to sample T2. For T2, there is a loss of strength as well as modulus, due to the hooping layers that are placed in the core of the composite sample. While the hoop layers are wound on the mandrel, the length of filament tows, consumed in one layer, is much smaller in T2. This ultimately reduces their contribution towards the bulk compression performance based on the Halpin–Tsai model. The load bearing performance is inferior, when all of the hoop layers are positioned one after another in case of T2, unlike in T1 and T3. Because the hoop layers do not get support from either side in T2, they are prone to fail under compressive load. There is higher chance of inter-layer delamination, which might be the reason for a substantially lower compressive modulus as compared to samples T1 and T3. When the hoop layers are surrounded by the helical layers on both sides, the best radial compressive behavior can be seen in the filament wound composite structures [27]. Though all of the samples were made from same type of tow and manufactured with five layers each, due to difference in winding pattern, there is a difference in radial compression behavior.

Further, it can be observed that the radial compressive strength as well as modulus is much higher in the case of tapered composite tubes as compared to cylindrical tubes. This might be attributed to compaction of the samples due to tapering by a small angle [21,27]. It is also supported by slightly higher fiber volume fractions in tapered samples, as can be seen in Table 2. The Halpin–Tsai model shows that with increasing fiber volume fraction, as well as an additional geometrical contribution of the tapering, the radial compressional performance is enhanced. The results of prediction based on this model show enhanced compressional performance in tapered samples T1 and T3.

**Figure 11:**
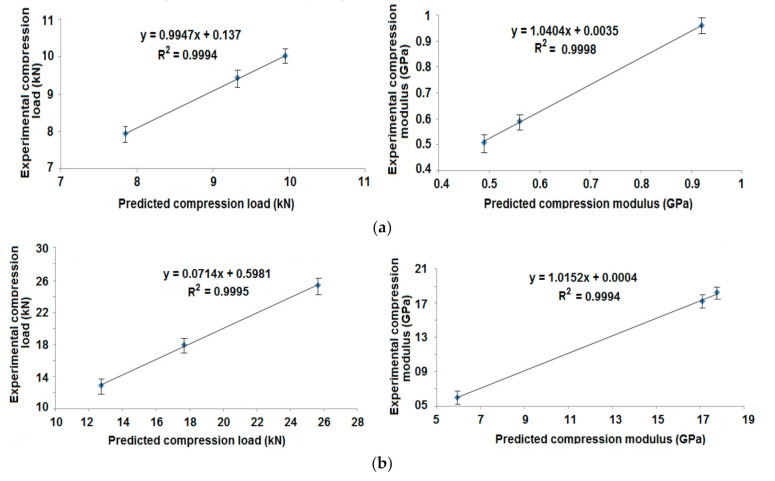
Correlation of experimental and predicted radial compression behavior of (**a**) Cylindrical composite samples at different angles of wind and (**b**) Tapered composite samples with different patterns of wind.

The experimental results of radial compression are compared with finite element-based prediction of these properties. The correlations are shown in Figure 11a,b for the cylindrical samples with different angles of wind and tapered samples with different patterns of winding, respectively.

The results of simulation/prediction were in agreement with the experimental findings. In most of the cases, the error was less than 5%. Thus, the prediction methodology was accepted to be appropriate for composite rocket launchers and the estimation of their radial compression performance.

#### 3.2.2. Axial Compression Behavior

**Figure 12:**
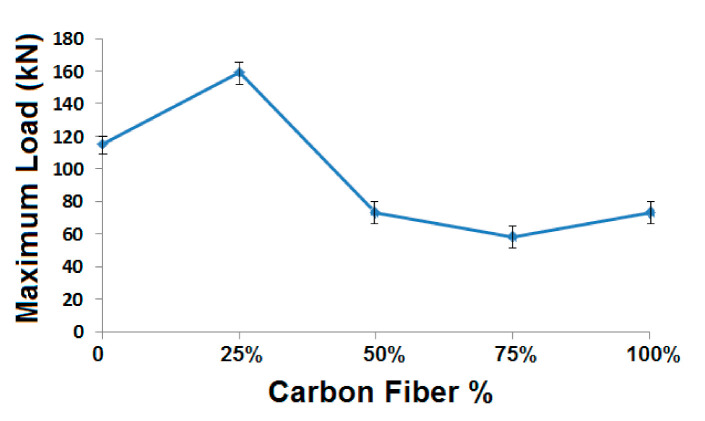
Axial compression load at different carbon fiber content.

Axial compression behavior of the composite samples was evaluated, as per ASTM standard. It is very much essential for understanding the performance and durability of composite rocket launcher, which undergoes substantial compression in the longitudinal (axial) direction following the propulsion of the rocket from the sleeve. Figure 12 shows the axial compressional behavior of the cylindrical composite samples with different glass and carbon fiber content. 

In general, it was observed that the axial compression performance is considerably superior to radial compression in all tubular composite samples that were developed through winding process [9]. It can be due to easy buckling/deformation in the radial direction, because of the fiber curvature. However, in the axial direction, the load is applied along in the perpendicular direction, i.e., perpendicular to fiber axis. The carbon and glass fiber tows are compact linear structures with fiber orientation in the coil direction. Thus, they offer much higher resistance to deformation in the thickness direction, which is the case of axial compression in the tubular samples. However, a radial compression is influenced by the curvature of the winding coils that offer much lower resistance to compressive deformation. These findings are supported by the results reported by several researchers [8,9].

In the case of axial compression, the trend was quite similar to radial compression with respect to the different carbon fiber%. Here, also, the sample with 25% carbon and 75% glass shows the highest compressive load. The sample with 75% carbon and 25% glass shows the minimum axial compressive load. The axial compression of the composite samples can be attributed as a complimentary behavior to the radial compression. As far as the hybrid samples are concerned, they follow the trend of the Halpin–Tsai model. The compressive strength decreases as the portion of carbon fiber tows increases, which has lower compressive strength when compared to glass tows. However, a deviation in the performance is observed in case of single fiber type samples. The compressive strength of sample with 25% carbon and 75% glass is even higher when compared to the compressive load bearing capacity of 100% glass-based sample. This behavior was also observed in case of radial compression. Thus, such a behavior is not just dependent on the direction of compression, but it is related to the bulk behavior that is governed by the interface of fibers with the matrix [29,31]. Because of a lower density of carbon fiber tows as compared to the glass fibers used, they occupy a slightly higher volume and at the same time present a higher interfacial surface area. This can be the reason for enhanced compressional strength when a relatively smaller proportion (25%) of carbon fibers are added in the sample. However, with a subsequent increase in carbon fiber%, the axial compressional performance deteriorates pertaining to lower compressional strength of carbon tows. 

In the case of 100% carbon based tubular composites, a very similar trend was observed under both radial as well as axial compression. Under axial compression mode, the compressive strength for 100% carbon-based sample was even higher than that of 50% carbon and 50% glass sample. This performance is rather more dominated by the geometrical arrangement of all-carbon tows than the fiber compressive properties themselves [33]. In an all-carbon fiber sample, there is an increased cohesiveness (due to absence of glass fiber tow), as was discussed earlier. At the same time, the carbon fiber tows present a higher interfacial area for bonding with matrix because of their relatively lower density. Thus, the mechanical performance, in general, and compressive property, specifically, is slightly enhanced. 

The hybridization of carbon (25%) and glass (75%) seems to attain an optimum balance with respect to inherent fiber property and the interfacial bonding. That is the reason for best performance with respect to both radial and axial compression with this hybrid combination. These observations reflect the importance of both mechanical and geometrical parameters, which are included in Halpin–Tsai equations [34].

**Figure 13:**
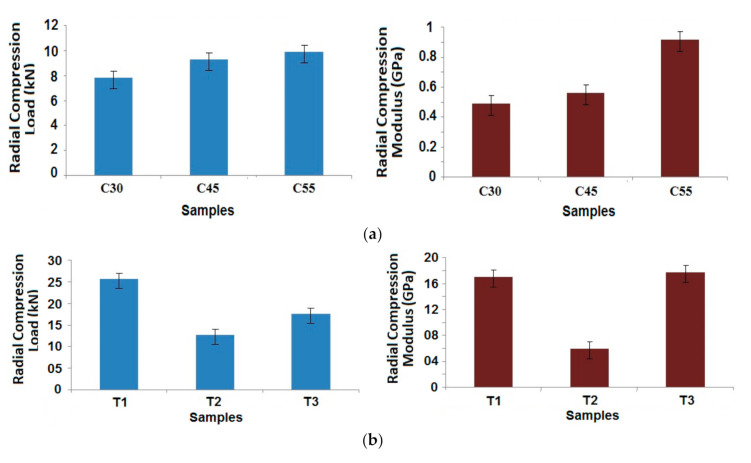
Axial compression behavior of (**a**) Cylindrical composite samples at different angles of wind and (**b**) Tapered composite samples for different patterns of wind.

Figure 13a,b show the axial compression test results for developed (25% carbon, 75% glass) samples for cylindrical samples at different angles of wind and tapered samples with different winding patterns, respectively.

Because the direction of loading is just opposite to radial compression, the outcomes are also just opposite in axial compression. Therefore, the sample with a lower helical angle is having higher compressional strength and modulus. With the lower angle of wind, the filaments are more oriented in the direction of loading. Thus, there is a higher contribution towards compressional resistance in the axial direction. Such behavior is in accordance with Halpin–Tsai equations due to higher contribution of the geometrical parameter towards the compressional performance [34,35]. 

In the case of tapered samples, it can be seen that the maximum axial load bearing capability is highest for the sample, having the hoop layers in between the helical layers. Actually, for both the axial and radial compressional behavior, the sample T1 is showing the best results among the tapered samples, as there is maximum interaction between the hoop and helical layers and the delamination is also minimized. 

On the other hand, samples T2 and T3 involve the aggregation of similar hooping/winding layers consecutively. This might lead to interlayer slippage/delamination during mechanical loading, e.g., under radial or axial compression. Furthermore, the smaller length of yarn coil in the hooping layers involves a lower fiber contribution in the middle layers, which undermines the bulk compressional property of the sample T2. The Halpin–Tsai model suggests the trend based on fiber property and geometry that is evident in the results obtained. The sample T3 performs better than T2 pertaining to the helical layers in the middle that are oriented better towards the load bearing direction. 

The axial compression behavior of cylindrical samples is superior when compared to tapered samples. It is because the loading is done in a direction that is perpendicular to fiber placement. A tapering is relatively unstable and might lead to fiber slippage during axial loading. This behavior is just opposite to radial compression behavior. Based on the geometry of tapering, the Halpin–Tsai model used in finite element analysis predicts a negative impact of taper angle on the axial compression behavior, while it positively influences the radial compressional performance [31,32,33].

**Figure 14:**
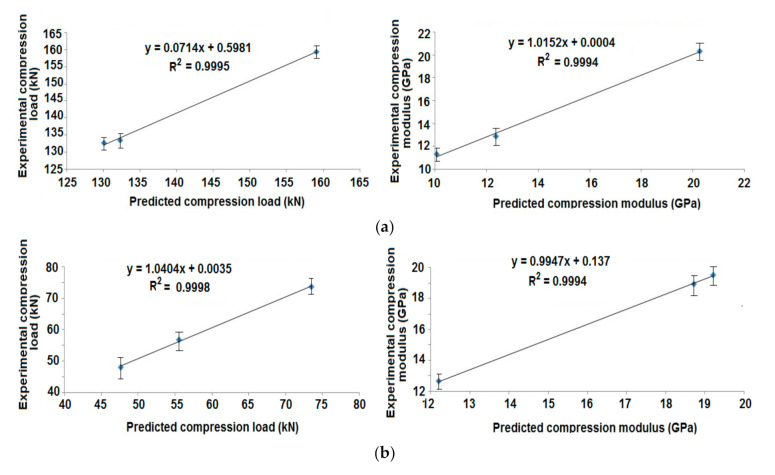
Correlation of experimental and predicted axial compression behavior of (**a**) Cylindrical composite samples at different angles of wind and (**b**) Tapered composite samples with different patterns of wind.

The experimental results of axial compression were compared with the predicted values of these properties. The correlation is shown in Figure 14a,b for cylindrical composite samples at different angles of wind and tapered composite samples with different patterns of wind, respectively.

The results of FEM based prediction show a very high correlation (R^2^ = 0.99) with the experimental findings. The error is less than 4%. Thus, the prediction is proved to be suitable for composite rocket launchers and the estimation of their axial compression performance. 

## 4. Conclusions

Robotized filament winding based tubular composite structures were developed from carbon and glass tows and epoxy resin by changing the sequence of stacking of hoop layers and helical layers and changing the angle of wind of the helical layers while keeping the sequence constant. Finite element analysis was used to predict the axial as well as radial compression behavior of composite tubes that are made by carbon and glass filament winding based on epoxy resin. The measured compressional properties of the tubular composites that were prepared by filament winding were compared with the predicted axial and radial compressional properties. The error percentage between the experimental and simulation results, in this case, was found to be very low, which means the model is accurate.

Filament wound tubular structures show reasonably good resistance to crush-in while the amount of carbon fiber is less than the amount of glass fiber in the hybrid structure especially with a 25:75 ratio of carbon to glass. The compression performance of glass rich tubular composites is superior to carbon rich composites following the Halpin–Tsai model based on fiber and matrix properties as well as filament winding geometry. Filaments in tubular composites, wound in some angle lower than 90°, show better compressional properties in both the axial and radial directions. Current research shows that the optimum angle is around 55°. Five layers of helical winding shows an optimum compressional behavior that can be acceptable for use in the specific product, e.g., Rocket-Propelled Grenade launchers (RPGs). The test standards for such composites that are used in RPGs can be based on NATO standards: STANAG-4526, AOP-4526, STANAG-4123, STANAG-4240, STANAG-4241, STANAG-4396, STANAG-4439, and STANAG-4440, etc. [14,15,16,17,18,19].

Several measurements are to be carried out and field testing has to finally be done as per military/NATO standards in order to make the samples ready for use in RPG launchers. Bench testing, like the hydraulic bursting test, hoop stress test, and several field testing, like leak proofing test, trial run for 1000 cycles, etc., must be carried out in order to determine the field performance.

## Figures and Tables

**Table 1:** Physical properties of used fibers and resin.

Properties	Glass	Carbon	Epoxy
Name	E-glass 6k	Torayca 12k	Araldyte LY556
Tensile strength (GPa)	3.44	3.53	0.345
Modulus (GPa)	73.5	230	25.5
Elongation %	4.8	1.5	1.2
Density (g/cc)	2.57	1.76	1.15
Compressive strength (GPa)	4.08	0.23	-
Viscosity (mPa. s)	-	-	10,000
Filament tow fineness (Tex)	600	600	-

**Table 2:** Details of composite samples developed.

Sample Name	Type	Angle of Wind (degrees)	Winding and Hooping Pattern	Wall Thickness (mm)	Fiber Volume Fraction
C30	Cylindrical	30	30-h-30-h-30	3.1	0.48
C45	Cylindrical	45	45-h-45-h-45	3.2	0.52
C55	Cylindrical	55	55-h-55-h-55	2.8	0.51
T 1	Tapered	45	45-h-45-h-45	2.7	0.51
T 2	Tapered	45	45-h-h-h-45	2.8	0.52
T 3	Tapered	45	h-45-45-45-h	3.1	0.52

## Data Availability

Not applicable. No data was reported.
